# PLGA Microspheres Loaded with β-Cyclodextrin Complexes of Epigallocatechin-3-Gallate for the Anti-Inflammatory Properties in Activated Microglial Cells

**DOI:** 10.3390/polym10050519

**Published:** 2018-05-11

**Authors:** Chun-Yuan Cheng, Quoc-Hue Pho, Xiao-Yu Wu, Ting-Yu Chin, Chien-Min Chen, Peng-Hsiang Fang, Yung-Chang Lin, Ming-Fa Hsieh

**Affiliations:** 1 Division of Neurosurgery, Department of Surgery, Changhua Christian Hospital, 135 Nanxiao St., Changhua City, Changhua County 500, Taiwan; 83998@cch.org.tw (C.-Y.C.); 96015@cch.org.tw (C.-M.C.); 2 Department of Biomedical Engineering, Chung Yuan Christian University, 200 Chung-Pei Rd., Chung Li District, Taoyuan City 32023, Taiwan; phoquochue@gmail.com (Q.-H.P.); venus801206@gmail.com (X.-Y.W.); 3 Center for Biomedical Technology, Chung Yuan Christian University, 200 Chung-Pei Rd., Chung Li District, Taoyuan City 32023, Taiwan; 4 Department of Bioscience Technology, Chung Yuan Christian University, 200 Chung-Pei Rd., Chung Li District, Taoyuan City 32023, Taiwan; tychin@cycu.edu.tw; 5 Division of Neurosurgery, Department of Surgery, Yuanlin Christian Hospital, No.456, Juguang Rd., Yuanlin City, Changhua County 510, Taiwan; doctorcombine@gmail.com; 6 Department of Veterinary Medicine, National Chung Hsing University, 145 Xingda Rd., South District, Taichung City 402, Taiwan

**Keywords:** microglia cells, epigallocatechin-3-gallate, PLGA, non-toxicity, biodegradable, anti-inflammation, bovine serum albumin, β-cyclodextrin

## Abstract

Although epigallocatechin-3-gallate (EG) is well-known as a potent antioxidant and free radical scavenger for neurodegenerative diseases, it still has disadvantages that reduce its treatment effectiveness due to low bioavailability, slow absorption, and water solubility. Therefore, the aim of this study is to improve the bioavailability of EG and increase the effectiveness of anti-inflammatory properties to microglial cells by using Poly(Lactide-*co*-Glycolide) (PLGA) microspheres as carriers. In this study, we used UV–Vis spectroscopy to show the formation of the complex of β-cyclodextrin (β-CD) and EG (CD-EG). The loading efficiency of EG in PLGA microspheres was optimized by the addition of β-CD. The highest loading efficiency of 16.34% was found among other formulations. The results of Fourier transform infrared spectroscopy indicated the loading of CD-EG in PLGA microspheres. The scanning electron microscopic images demonstrated the spherical PLGA particles with controlled particles size ranging from 1–14 µm. Moreover, the in vitro release of EG was conducted to explore the sustained release property of the PLGA formulations. In the in vitro model of mouse microglial cells (BV-2 cells) stimulated by lipopolysaccharide, the cytotoxicity test showed that for up to 1 mg/mL of PLGA microspheres no toxicity to BV-2 cells was found. PLGA microspheres can significantly suppress the nitric oxide production from BV-2 cells, indicating EG loaded in PLGA microspheres can suppress the inflammation of activated microglial cells. Furthermore, the intracellular iNOS in BV-2 cells was also found to be down regulated. In summary, we have successfully shown that the use of β-CD can increase the loading efficiency of EG in PLGA microspheres and provide neuroprotective effect on the activated microglial cells.

## 1. Introduction

Microglia are the primary immune cells of the brain that play a crucial role in neurodegenerative diseases [1,2]. In regards to brain injury caused by toxic reagents, trauma, ischemia, and cell debris, the microglial cells are stimulated to generate excessive factors of pro-inflammation consisting of tumor necrosis factor (TNF-α) [3,4], interleukin (IL-1β) [5,6], nitric oxide (NO) [7,8], and reactive oxygen species (ROS) [9] to induce death of neurons. Activated microglia is described to present in two functionally various states, including innate and adaptive activation, which is determined by their stimulatory environment. The inequality of these activation in microglial cells may lead to either neural benefit or damage. The activation of microglial cells in terms of chronicity is believed to be related to neurodegenerative disorders (Alzheimer’s disease [10,11,12] and Parkinson’s disease [13,14,15]). Hence, the inhibition of microglial activation has been suggested for potential treatment of neurodegenerative diseases [16,17].

In recent years, epigallocatechin-3-gallate (EG), one of the most active polyphenols in green tea leaves, has been the subject of significant research owing to its numerous therapeutic benefits in many diseases, including cardiovascular disorders [18], obesity [19,20] and cancer [21], as well as to slowing down the process of aging [22]. Especially, it has been well-known as an antioxidant [23], as well as an anti-inflammatory agent [24] that might inhibit microglial activation inducing neural degeneration of the brain in Parkinson’s disease [25]. EG has several advantages, such as anti-inflammation, antioxidant activity, and the reduction of neural cell death [26]. The neuroprotective effect of EG to microglial cells could be explained by a variety of mechanisms, such as the down-regulation of pro-apoptotic genes, promotion of cell survival, defense against anti-inflammation, and scavenging of ROS [27]. EG has been also demonstrated to directly scavenge reactive oxygen species to reduce inflammation. The antioxidant effects of EG is related to hydroxyl groups of the phenolic groups in the chemical structure of EG. Particularly, the phenolic groups of EG work as electron donors that can transfer hydrogen atoms or a single electrons. Additionally, the phenolic molecule is able to make an internal modification to stabilize the unpaired electron after the donation of electrons. EG can potentially suppress the release of NO and TNF-α from LPS-induced microglial activation through the down-regulation of iNOS and TNF-α gene expression. Although EG has plenty of benefits with respect to antioxidant and anti-inflammatory effects, it still has some disadvantages that reduce the therapeutic effect due to poor bioavailability [28], water solubility [29], slow absorption, fast metabolism, and elimination. 

In order to improve the bioavailability of EG with longer circulation, poly lactic-*co*-glycolic acid (PLGA) was used as a biodegradable and biocompatible carrier [30,31] to deliver EG to increase the effectiveness of anti-inflammatory properties in mouse microglial cells. It is approved as a family of biodegradable polymers which are physically strong and highly biocompatible. PLGA has been studied as delivery vehicles for drugs, proteins, and various other macromolecules, such as DNA [32], RNA [33], and peptides [34]. Among various available biodegradable polymers, PLGA is the most broadly used polymer for its advantages, such as a long history of clinical application, favorable degradation characteristics, and sustained delivery of drugs.

Although PLGA is biodegradable and biocompatible, which are required functions for drug delivery systems, the solubility of EG in PLGA is relatively low. To overcome the drawback, two strategies could be considered: First, hydrophilic carriers, such as serum proteins, could be used. Second, inclusion compounds, such as cyclodextrins, could be implemented. Serum albumin is known as waste and nutrient carriers in blood pool in human. EG was found to bind to some cellular proteins [35]. Ovalbumin, a protein in egg white, was reported to co-bind EG with polysaccharides to fabricate nanoparticles to resist gastrointestinal digestion [36]. Other than macromolecules, like albumin, oligo-saccharides, such as cyclodextrins (CDs), are known to complex various compounds in their hydrophobic cavities, and the inclusion complexes can lead to alterations of the physical, chemical, and biological properties of the guest molecule. Among cyclodextrins, β-CD is the most widely used compound due to the optimal size of its internal cavity (6–6.5 Å) for the encapsulation of EG. Ishizu et al. reported that EG can interact with β-CD in aqueous solution to form the inclusion complex via van der Waals, hydrophobic, and hydrophilic interactions [37]. These interactions lead to an enhanced stability of EG that is even more pronounced in the case of β-CD derivatives as they contain side chains in the rim region for further interactions [38].

In the present study, β-CD was chosen to interact with EG to form a very stable complex to improve the loading efficiency when encapsulated into PLGA microspheres. The cytotoxicity and the in vitro anti-inflammatory assays of activated BV-2 cells were conducted in the presence of PLGA-CD-EG formulations.

## 2. Experimental Section

### 2.1. Materials

Polyvinyl alcohol (PVA, 130,000 Da), dimethyl sulfoxide (DMSO), potassium bromide (KBr), chloroform were purchased from Echo Chemical Co., Ltd. (Toufen, Miaoli County, Taiwan). 3-(4,5-dimethylthizol-zyl)-2,5-diphenyl tetrazolium bromide (MTT reagent), β-cyclodextrin, bovine serum albumin, sodium nitrite (NaNO_2_), lipopolysaccharide (LPS), Griess reagent, Roswell Park Memorial Institute (RPMI) 1640 medium (RPMI-1640), and the TNF-α ELISA kit, were purchased from Sigma-Aldrich Co., Ltd. (St. Louis, MO, USA). Three different PLGAs were obtained from Green Square Materials Inc. Co., Ltd. (Taoyuan City, Taiwan), e.g., PLGA 75/25 with the inherent viscosity (I.V) of 0.85 dL/g, PLGA 75/25 with I.V of 0.53 dL/g, and PLGA 50/50 with I.V of 0.49 dL/g, symbolized as PLGA_0.85_, PLGA_0.53_, and PLGA_0.49_, respectively. (−)-Epigallocatechin-3-gallate (EG, purity of 85%) was extracted using green tea leaves of Tenren‘s tea company (Taipei, Taiwan) [39].

### 2.2. Preparation of Epigallocatechin-3-Gallate-β-Cyclodextrin (CD-EG) Complexes

EG and β-CD were dissolved in water with different concentrations (20, 40, 50, 60, 80 µM). The reaction was carried out at room temperature by mixing solutions together using a vortex for 30 min. After reaction, free EG was removed from the complex by using a dialysis bag (MWCO = 2000 Da, Spectra/Por^®^ 6 Dialysis Tubing (Spectrum Labs, Fort Worth, TX, USA) against 2 L of H_2_O for one day. The samples were then lyophilized to give dry powder. The feeding ratio of β-CD to EG was shown in Table 1. The absorption intensity and wavelength of the dried complexes was determined by UV–Vis spectroscopy from 200–600 nm.

### 2.3. Preparation of Epigallocatechin-3-Gallate-β-Cyclodextrin Complex-Loaded PLGA Microspheres

In this study, the synthesis of PLGA microspheres was carried out for loading EG with some modifications by using a water-in-oil-in-water emulsion procedure with the solvent extraction technique [40]. The feeding amounts of CD-EG, BSA-EG or EG in PLGA matrix are listed in Table 2. The abbreviations of the samples are also listed in the table. Briefly, different amounts of CD-EG (0, 1, 5, 10, 15 mg) were dissolved into an amber vial containing 1 mL of PBS solution (pH = 7.4) and then 250 mg of PLGA was dissolved into 10 mL of chloroform solvent in a vial under overnight stirring to ensure PLGA completely dissolved in the solvent. Later, 1% polyvinyl alcohol (PVA) solution was prepared by adding 0.5 g of PVA into 50 mL of hot water (90 °C) and the EG solution was slowly transferred into the PLGA solution. The mixture was homogenized twice for 5 sec with a 10 sec pause in between to obtain the first emulsion. Immediately, 10 mL of PVA solution was pipetted into the first-degree emulsion. The homogenizer was turned on, and the power gradually turned up until mixing took places, and it was allowed to run continuously for 15 min to achieve the second-degree emulsion. Then, the second-degree emulsion was transferred into a 100 mL Erlenmeyer flask covered with aluminum foil and containing 40 mL of PVA solution. The mixture was regularly mixed overnight with a magnetic stirrer bar at 800 rpm to evaporate the solvent and to keep the emulsion stable. In order to collect particles, the obtained microspheres were centrifuged and washed five times with ddH_2_O at 1600 rpm. The dry microspheres were achieved by freeze drying.

### 2.4. Crude Yield and Loading Efficiency

In order to determine the amount of CD-EG in PLGA microspheres, 1 mg of microspheres was dissolved into 1 mL of DMSO solvent and analyzed by UV–Vis spectrometry at 266.5 nm. The concentration of CD-EG in PLGA was computed by using the standard curve of CD-EG based on various concentrations and absorbance (optical density), where *W*_CD-EG_ is the real CD-EG concentration and *W*_PLGA-CD-EG_ is the given concentration of PLGA-CD-EG, respectively. The drug loading efficiency (DLE), as a percentage, was calculated using Equation (1):

(1) $$\mathrm{DLE}\;\left(\%\right)=\frac{W_{\mathrm{EG}-\mathrm{CD}}}{W_{\mathrm{PLGA}-\mathrm{CD}-\mathrm{EG}}}\times 100\%$$

### 2.5. Fourier Transform Infrared Spectrometry (FTIR)

After the fabrication of materials, CD-EG complex-loaded PLGA was investigated by FTIR to determine the typical functional groups that are present in the chemical structure of the materials. Briefly, a small amount of each material was mixed well with KBr salt (ratio 1:100). Then, the samples were compressed into membranes by using a hydraulic press. Lastly, the membranes were transferred into the FTIR spectrometer for characterization.

### 2.6. Morphology of CD-EG Complex-Loaded PLGA Microspheres

After preparation of CD-EG complex-loaded PLGA microspheres, the resulting samples were investigated under SEM (JSM-6300, JEOL, Tokyo, Japan). In brief, the samples are mounted on a stub of metal with adhesive, coated with 40–60 nm of metal, such as gold/palladium, and then observed in the microscope. Samples were observed on a field emission gun SEM under low vacuum mode at a tension ranging to about 5 kV. The average particle size and the size distribution of the PLGA microspheres were calculated by a freeware of Java image processing program (Image J, National Institute of Health, Bethesda, MD, USA)

### 2.7. Release Profile of CD-EG Complex-Loaded PLGA Microspheres

Ten milligrams (10 mg) of microspheres were immersed into a centrifugation tube containing 5 mL of PBS solution (pH 7.4). Then, the tube was continuously shaking with 500 rpm and incubated at 37 °C to maintain the environmental temperature. After different time intervals, the mixture was centrifuged at 2000 rpm at 25 °C for 10 min to ensure all microspheres aggregate at bottom of the centrifugation tube. Then, 1 mL of solution was pipetted to determine the real CD-EG concentration by using a UV–Vis spectrometer at 266.5 nm. Finally, 1 mL of fresh PBS was replaced back into the mixture. The experiments were repeated in triplicate. The cumulative percentage release and released concentration was calculated using Equations (2) and (3):

(2) $$\mathrm{Amount}\;\mathrm{of}\;\mathrm{drug}\;\mathrm{released}\;\left(µg/\mathrm{mL}\right)=\frac{\left[\mathrm{drug}\right]\times \mathrm{volume}\;\mathrm{of}\;\mathrm{dissolution}\;\mathrm{bath}\times \mathrm{dilution}\;\mathrm{factor}}{1000}\times 100$$

(3) $$\mathrm{Cumulative}\;\mathrm{percentage}\;\mathrm{release}\;\left(\%\right)=\frac{\mathrm{Sampling}\;\mathrm{volume}\;\left(\mathrm{mL}\right)}{\mathrm{Bath}\;\mathrm{volume}\left(\mathrm{mL}\right)}\times P_{\left(t-1\right)}+P_{t}\;100\%$$

where the concentration of CD-EG (µg/mL) was determined using the standard curve of CD-EG in water based on UV–Vis spectroscopy at a wavelength of 266.5 nm. *P*_t_ is the percentage release at time t and *P*_(t−1)_ is the percentage release in the previous sampling time.

### 2.8. BV-2 Cell Culture

The BV-2 microglial cell line was obtained and characterized as described [41]. Cells were cultivated in RPMI containing 10% heat-inactivated fetal ovine serum supplemented with l-glutamine (4 mM) and gentamicin 5 (μg/mL) in H_2_O-saturated 5% CO_2_ atmosphere at 37 °C. BV-2 microglia were maintained without mycoplasma contamination.

### 2.9. Cytotoxicity

The BV-2 microglia cells were seeded at a density of 1 × 10^4^ cells per well for 24 h. The medium from the previous culture was removed and replaced with fresh medium. Cells were incubated with different formulations of drug-loaded PLGA microspheres for 24 h. After incubation, the medium was removed and washed once with PBS. The medium and MTT reagent in a volume ratio of 9:1 mixed was added into BV-2 cells and incubated for 3 h. The medium was removed after the reaction. Then, DMSO was added to dissolve the purple crystals and reacted for 5 min. An enzyme immunoassay analyzer was used to detect the absorption wavelength of 570 nm of each well. The cell viability formula is as follows (Equation (4)):

(4) $$\mathrm{Cell}\;\mathrm{viability}\;\left(\%\right)=\frac{{\mathrm{Absorbance}}_{\mathrm{sample}}}{{\mathrm{Absorbance}}_{\mathrm{control}}}\cdot 100\%$$

### 2.10. NO Qualification

In order to determine quantitatively the NO production from microglial cells, the accumulation of NO_2_^−^ was investigated as an indicator using the Griess reaction assay. In brief, BV-2 microglial cells 1 × 10^5^ (cells/well) were seeded in each well of 96-well plates and kept overnight. Later on, BV-2 cells were then transferred to phenol-red free DMEM. BV-2 cells then were pre-treated with various prepared formulations of PLGA microspheres under incubation for 24 h. Later on, BV-2 microglial cells were further incubated for 24 h with LPS (50 ng/mL). After 24 h, equivalent volumes of Griess reagent were added to BV-2 cell supernatants and then incubated at room temperature for 20 min. Nitrite concentrations were determined by using standard solutions of sodium nitrite prepared in cell-culture medium. The absorbance at 540 nm was determined using an ELISA reader at room temperature. Each experiment was performed in triplicate.

### 2.11. Western Blot

One milligram (1 mg) of PLGA_0.49_-(CD-EG) formulation and 50 μg of protein were separated on 10% SDS-PAGE (sodium dodecyl sulfate-polyacrylamide gel electrophoresis) and transferred to PVDF (polyvinylidene difluoride) membranes. Later on, the samples were incubated for 1 h with 5% dry skim milk in TBST buffer at room temperature to prevent nonspecific binding. Next, the resulting samples were then incubated with rabbit anti-iNOS (1: 1000). Lastly, the resulting samples were incubated with alkaline-phosphatase-conjugated goat anti-rabbit secondary antibody at a ratio of 1: 1000 (Alexa Fluor^®^ 488 goat anti-rabbit IgG, Molecular Probes, Eugene, OR, USA) for 1 h at room temperature. Bands were visualized using the chromogenic substrate 5-bromo-4-chloro-3-indolyl phosphate in the presence of nitroblue tetrazolium.

### 2.12. Statistical Analyses

The data are presented as the mean ± SD and were analyzed using Origin 8.0 software (Originlab Northampton, MA, USA). The groups were compared by one-way analysis of variance (ANOVA) followed by the least significant difference test. A *p* value of less than 0.05 was considered statistically significant.

## 3. Results and Discussion

### 3.1. Preparation of Epigallocatechin-3-Gallate-β-Cyclodextrin (CD-EG) Complexes

**Figure 1:**
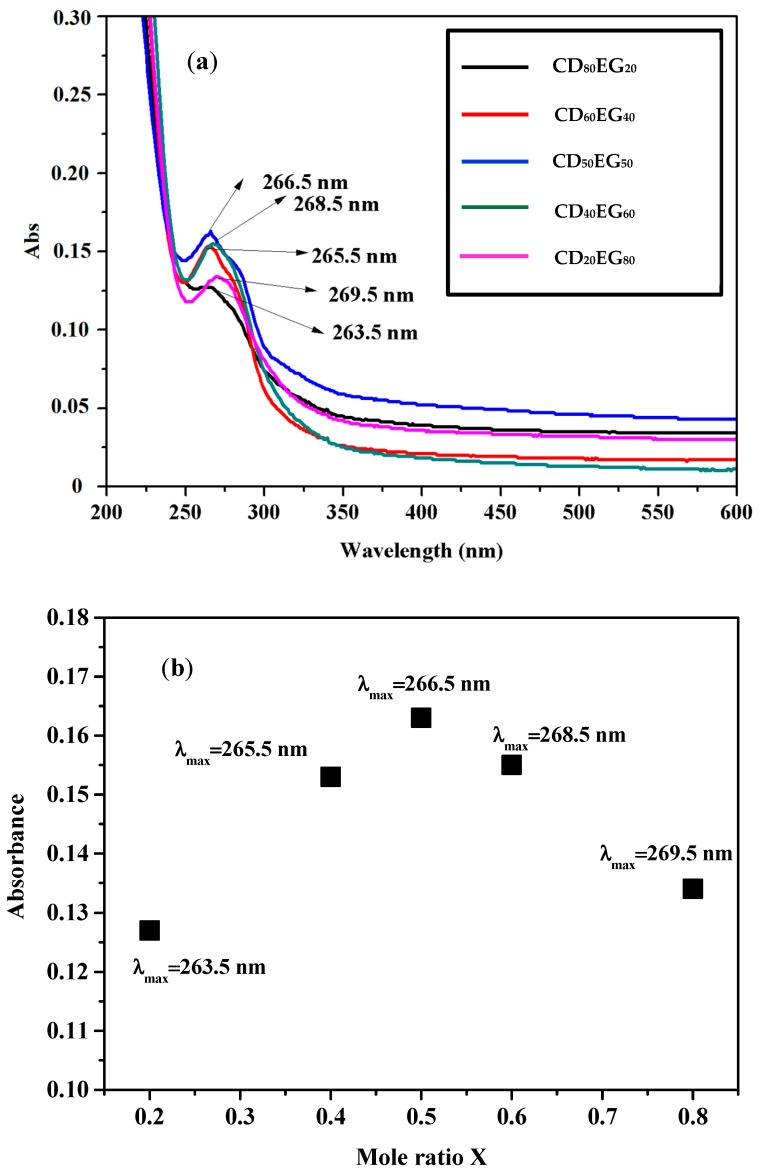
(**a**) UV–Vis spectra of CD-EG complexes at various concentrations at wavelength 200–600 nm; (**b**) Plots of the CD-EG inclusion complex where the mole ratio *X* = [EG]/([EG] + [CD]) at total concentration [EG] + [CD ] = 100 µM. The y-axis corresponds to the absorbance of the shifting peak of CD-EG compared to initial absorbance of un-reacted EG (274 nm) at 25 °C.

Preparation of CD-EG complexes has been previously reported [37]. As known, EG can interact with β-CD via van der Waals, hydrophobic interactions, and hydrophilic interaction. In this study, UV–Vis spectroscopy was used to prove the interaction between EG and β-CD. Figure 1 shows the UV–Vis spectra of CD-EG complexes and the maximum intensities of the CD-EG complexes with respect to their absorption wavelengths.

As observed in Figure 1a, the absorption maximum for different CD-EG complexes varied with respect with the mole ratio *X* of feedings of CD and EG. The result demonstrated the influence of β-CD to the absorption peak of EG. In other words, these results revealed that various ratios of β-cyclodextrin interacted with EG lead to the shifting of maximum adsorption wavelength of complexes compared to original EG (274 nm) [42]. In particular, the maximum adsorption wavelength shifted from the original wavelength of EG (274 nm) to 269.5, 268.5, 266.5, 265.5, and 263.5 nm corresponding to EG_80_CD_20_, EG_60_CD_40_, EG_50_CD_50_, EG_40_CD_40_, and EG_20_CD_20_, respectively. From those UV–Vis results, it demonstrated that EG can interact with β-CD to form the inclusion complexes.

As can be seen in Figure 1b, when the mole ratio *X* increased from 0.2 to 0.8, the wavelength for maximum adsorption of CD-EG complexes shifted from 263.5 to 269.5 nm. However, the intensity of the maximum adsorption increased from 0.127 to 0.163 when the mole ratio *X* increased from 0.2 to 0.5. When the mole ratio *X* increased from 0.5 to 0.8, the intensity of the maximum adsorption decreased. The result demonstrated that the mole ratio *X* of 0.5 resulted in the highest intensity of maximum adsorption, meaning that EG can interact with β-CD at the ratio of 1:1 [37]. We found that UV–Vis spectroscopy could be used to determine the optimized mole ratio *X* of CD-EG reaction, which is coincident to previous investigations of ^1^H-NMR on the CD-EG complex [43]. In the present study, the complex EG_50_CD_50_ was optimized for further experiments.

### 3.2. Preparation of CD-EG Complex-Loaded PLGA Microspheres

CD-EG complex-loaded PLGA microspheres were prepared using the double-emulsion (water-in-oil-in-water) evaporation technique. In this study, β-CD was used to form inclusion complexes with EG to improve their loading efficiency into PLGA microspheres due to low bioavailability and water-soluble properties of EG that decrease treatment efficacy. In this case, PLGA was dissolved in chloroform as the hydrophobic phase, while CD-EG dissolved in PBS and PVA solution functioned as the first and second hydrophilic phases. In order to prove the alternative of β-CD to improve the loading efficiency of EG into PLGA microspheres, other various formulations were prepared to make a comparison. The results revealed that PLGA_0.49_ performed as the most potential material to prepare PLGA microspheres, compared to PLGA_0.53_ and PLGA_0.85_. In particular, using PLGA_0.49_ resulted in more stable and single microspheres, while PLGA_0.53_ and PLGA_0.85_ caused to the aggregation of individual particles. In addition, the dried PLGA_0.53_ and PLGA_0.85_ were more difficultly dispersed in water than that of PLGA_0.49_. This phenomenon was due to the balance of intermolecular hydrophilic and hydrophobic phases of PLGA. For PLGA_0.49_, this was explained as the balance of intermolecular hydrophilic and hydrophobic interactions (lactic acid:glycolic acid, 50:50), leading to the stability of microspheres.

### 3.3. FTIR Characterization

**Figure 2:**
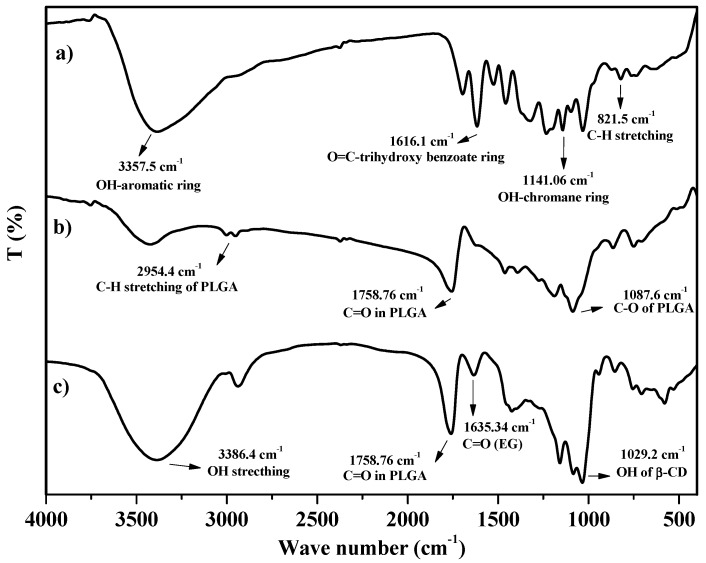
FTIR spectra of (**a**) EG; (**b**) PLGA-EG; and (**c**) PLGA-CD-EG.

To demonstrate the entrapment of PLGA polymer on CD-EG complexes, the prepared samples were tested by FTIR to determine functional groups of compounds existing in the samples. The result in Figure 2 shows the FTIR spectrum of pure EG, PLGA-EG, and PLGA-CD-EG samples. For the FTIR spectrum of EG (Figure 2a), the appearance of typical adsorption peaks was demonstrated for specific functional groups of EG. In particular, the adsorption band at 3357.5 cm^−1^ indicated OH groups linked to aromatic rings. Moreover, specific peaks at 1616.1, 1141.06, and 821.5 cm^−1^ demonstrated C=O groups connected to the trihydroxy benzoate ring, OH groups linked to the chromane ring, and C–H stretching, respectively [44]. For the FTIR spectrum of PLGA-EG (Figure 2b), the results showed that there were new typical peaks that revealed the representation of the PLGA polymer in PLGA-EG. In detail, some new specific adsorption bands appeared at 2954.4, 1758.76, and 1087.6 cm^−1^, indicating C-H stretching, C=O groups, and C–O stretching, respectively [45]. In addition, some typical peaks of EG also appeared in the spectrum. The results proved that PLGA-EG components included PLGA and EG. For the FTIR spectrum of PLGA-CD-EG (Figure 2c), the result also revealed typical peaks of PLGA and EG. Apart from that, there were some specific adsorption bands describing for the existence of β-CD. For examples, the absorption peaks at 3386.4 and 1029.2 cm^−1^ indicated for β-CD [46]. In short, the FTIR spectrum revealed the functional groups of each component in PLGA-CD-EG.

### 3.4. Loading Efficiency

**Figure 3:**
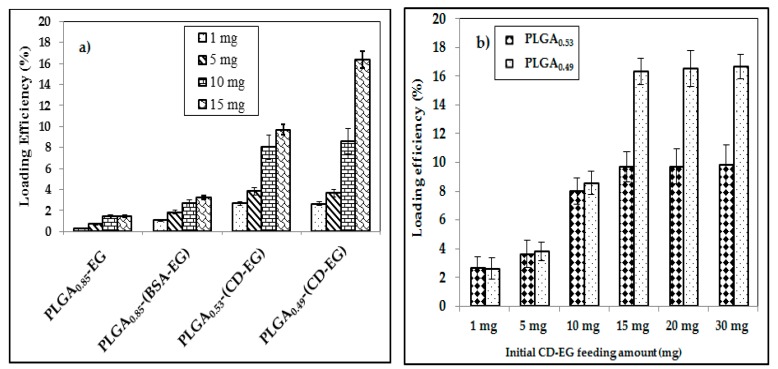
(**a**) Loading efficiency of various formulations of PLGA microspheres with different EG feeding amounts; and (**b**) optimization of the effects of the initial EG feeding amount on the loading efficiency of PLGA_0.53_-(CD-EG) and PLGA_0.49_-(CD-EG).

In this study, various micro-particle formulations of PLGA were fabricated using different materials, such as bovine serum albumin (BSA) and β-CD, to improve the loading efficiency. The loading efficiency of EG was calculated with Equation (2). The results are shown in Figure 3a and Table 3. As one can see, PLGA_0.53_-(CD-EG) and PLGA_0.49_-(CD-EG) resulted in much higher loading efficiencies than PLGA_0.85_-EG and PLGA_0.85_-(BSA-EG). PLGA_0.85_, with a loading efficiency of 0.26–2.06%, is not suitable for loading EG due to low loading contents and ease of aggregation [47]. Even though BSA has been reported to form complexes with EG [35], the loading efficiency of PLGA_0.85_-(BSA-EG) is still low with a loading efficiency of 1.07–3.24%. In the present study, PLGA_0.85_-(BSA-EG) was prepared as follows: Firstly, EG solution (water phase) was dispersed in oil phase (chloroform) by a homogenizer to generate W/O emulsion. Continually, the first W/O emulsion was dispersed into PVA (another water phase) under stirring overnight. Therefore, EG has the tendency to move out from the first water phase, leading to a low efficiency of encapsulation. This is different from the binding of cellular proteins with EG intracellularly, as reported in [35].

Compared with PLGA microspheres containing BSA, PLGA_0.49_-(CD-EG) and PLGA_0.53_-(CD-EG) had loading efficiencies of 2.68–16.34% and 2.64–9.69% (Figure 3b), displaying the role of β-CD in the dissolution of EG in PLGA microspheres. This is due to the chemical structure of β-CD with hydrophilic and hydrophobic phases in the outer and inner surfaces [48]. Therefore, β-CD could function as an interface between EG and PLGA through hydrophilic and hydrophobic interactions.

We further examined PLGA_0.49_-(CD-EG) formulations. When increasing the feeding amount of CD-EG complexes, the loading efficiency significantly increased from 2.68–16.34%. Among them, PLGA_0.49_-(CD-EG)_15_ with 15 mg of feeding CD-EG resulted in the highest loading efficiency (16.34%). Compared to previous reports for EG loaded PLGA nanoparticles (5.76%) [40], the present study demonstrated that β-CD could significantly improve the loading efficiency of EG in PLGA microspheres (16.34%). When further increasing the feeding of CD-EG from 15 to 30 mg, the loading efficiency of EG remained unchanged or insignificantly increased. This demonstrated that the loading capacity was mostly saturated when the initial feeding amount of CD-EG reaches 15 mg.

### 3.5. Morphology, Particle Size, and Size Distribution

**Figure 4:**
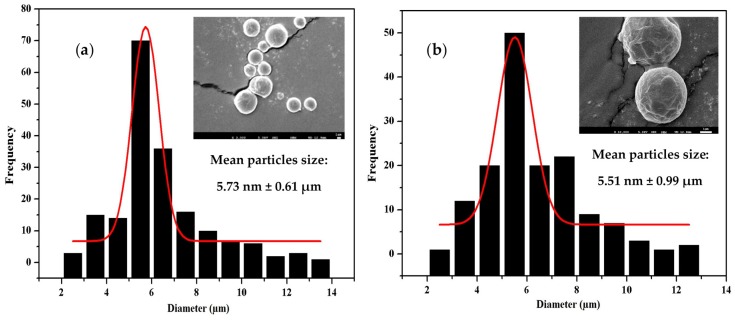

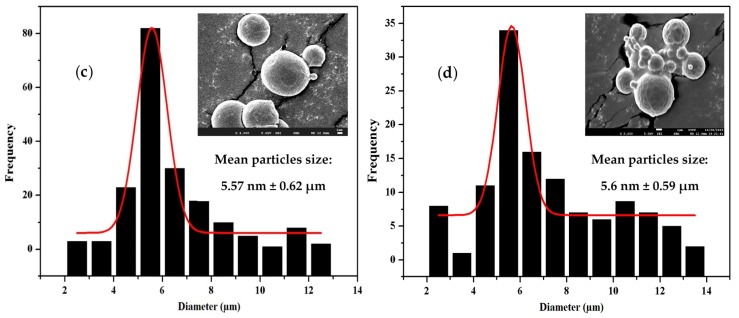
Morphology, particles size, and distribution of (**a**) PLGA_0.49_-(CD-EG)_0_, (**b**) PLGA_0.53_-(CD-EG)_15_, (**c**) PLGA_0.49_-(CD-EG)_15_, and (**d**) PLGA_0.85_-EG_15._

To determine the particle size and distribution of PLGA microspheres before and after CD-EG loading, various formulations of PLGA microspheres were investigated by SEM. Figure 4 shows the average particle size and distribution of microspheres determined by Image J software. As seen, the average particle sizes of PLGA_0.49_-(CD-EG)_0_ (Figure 4a), PLGA_0.53_-(CD-EG)_15_ (Figure 4b), PLGA_0.49_-(CD-EG)_15_ (Figure 4c), and PLGA_0.85_-EG_15_ (Figure 4d) were 5.73 ± 0.61, 5.51 ± 0.99, 5.57 ± 0.62, and 5.6 ± 0.59 µm, respectively. These results proved that the average particle size of PLGA microspheres was insignificantly changed. Therefore, it can be concluded that CD-EG did not influence to morphology and average particle size of PLGA microspheres. In addition, the results in Figure 4 also revealed that the distribution of particle sizes of PLGA_0.49_-(CD-EG)_0_, PLGA_0.85_-EG_15_, PLGA_0.53_-(CD-EG)_15_, and PLGA_0.49_-(CD-EG)_15_ all ranged from 1–14 µm. These results demonstrated that PLGA micro-particles still distributed well after CD-EG loading.

As observed, the SEM results showed that the on surface of microspheres is smooth, with a spherical shape. In addition, the SEM results showed that the particle size of various PLGA formulations was distributed from 1–14 µm. These results proved that there was no significant difference in particle size of PLGA microspheres using the double-emulsion evaporation technique even though CD-EG was loaded into the particles. Apart from this, the SEM results also indicated the aggregation of PLGA microspheres after freeze drying, as shown in Figure 4d. This was explained by the formation of ice crystals and hydrophobic properties of PLGA_0.85_ possibly causing aggregation of the microspheres, which leads to the reduction of PLGA micro-particle resuspension [49]. One more time, the results of SEM demonstrated the success of the preparation of PLGA microspheres CD-EG complexes with spherical shape and micron-scale size.

### 3.6. Release Profile

**Figure 5:**
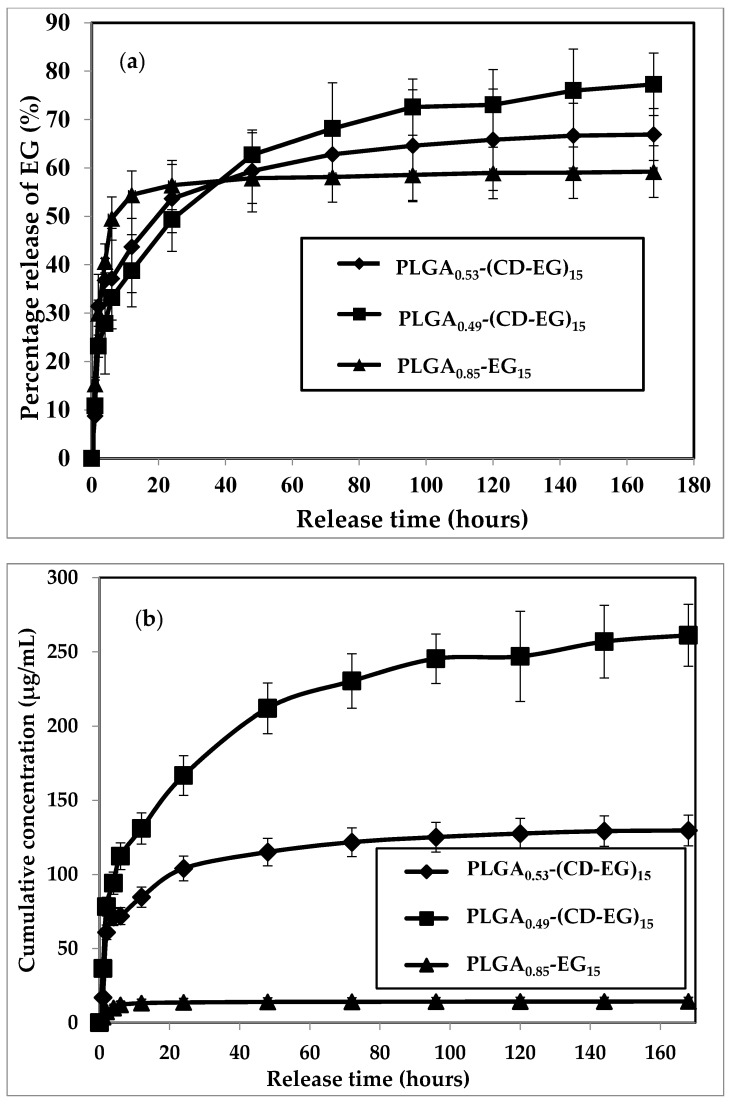
Profiles of percentage release (**a**) and cumulative concentration (**b**) of EG from various PLGA microspheres in PBS (pH = 7.4, 37 °C, shaking speed at 500 rpm).

The release of EG from PLGA microspheres loaded with/without CD-EG complexes were experimented in PBS solution (pH 7.4) incubated at 37 °C. The results are shown in Figure 5. The percentage release of EG from PLGA_0.85_-EG_15_, PLGA_0.53_-(CD-EG)_15_, and PLGA_0.49_-(CD-EG)_15_ versus the release time is illustrated in Figure 5a, and the cumulative amount of released EG is shown in Figure 5b.

In Figure 5a, the release profile of EG consists of two phases: the initial release and sustained release. In terms of the time for 50% of EG released from the PLGA microspheres, PLGA_0.85_-EG_15_ showed the initial release of 50% EG at the sixth hour, whereas the times for 50% EG released from PLGA_0.53_-(CD-EG)_15_ and PLGA_0.49_-(CD-EG)_15_ were 20 and 24 h, respectively. A sign of the burst release of EG from PLGA_0.85_-EG_15_ is explained by EG being relatively water-soluble as compared to the hydrophobic PLGA polymer. Therefore, low affinity of EG to PLGA led to the quick release of EG into PBS solutions. In terms of the total amount of EG released, 59.24% of EG was released from PLGA_0.85_-EG_15_, while that of PLGA_0.53_-(CD-EG)_15_ and PLGA_0.49_-(CD-EG)_15_ were 66.91% and 77.27%, respectively. The significant difference of release profiles among the three PLGA microspheres can be attributed to the function of β-CD in controlling the release rate of EG.

In Figure 5b, in terms of the cumulative amount of EG released at the end of the release experiment, it is obvious that PLGA_0.85_-EG_15_ can only release 14.33 μg/mL of EG. Conversely, PLGA_0.49_-(CD-EG)_15_ and PLGA_0.53_-(CD-EG)_15_ can release 261.17 μg/mL of EG and 129.67 μg/mL of EG, respectively. Such a significant difference in these two PLGA microspheres could be attributed to the ratio of LA and GA, where PLGA_0.49_ has a ratio of LA:GA = 50:50 and PLGA_0.53_ has a ratio of LA:GA = 75:25. There were reports for the effect of the LA/GA ratio on the hydrolysis of PLGA [50]. The more GA segment in the PLGA, the faster the hydrolysis can proceed. Since PLGA with LA:GA = 50:50 is widely known as the fastest hydrolyzed polymer [51], PLGA_0.49_-(CD-EG)_15_ released more EG indicating the hydrolysis is the driving force of the release of EG from PLGA microspheres.

Accordingly, here are the factors influencing the release profiles: 1. diffusion, 2. intermolecular interaction between EG and β-CD; and 3. hydrolysis (affected by LA/GA ratio of PLGA). Apparently, diffusion is the major driving force for passive drug delivery systems (PLGA microspheres in the present study). However, β-CD, introduced to increase more EG dissolved in PLGA, was also found to affect the initial release of EG (Figure 5a) in the first 24 h of release experiment. When the release time was extended to 168 h (seven days), the hydrolysis of PLGA was accompanied by the release of EG. Since the hydrolysis of PLGA is determined by the LA/GA ratio, it turned out that the LA/GA ratio could greatly control the total amount of EG released from the PLGA microspheres (Figure 5b). This also concludes the biphasic release profiles as mentioned in the beginning of this section.

### 3.7. Cell Viability

**Figure 6:**
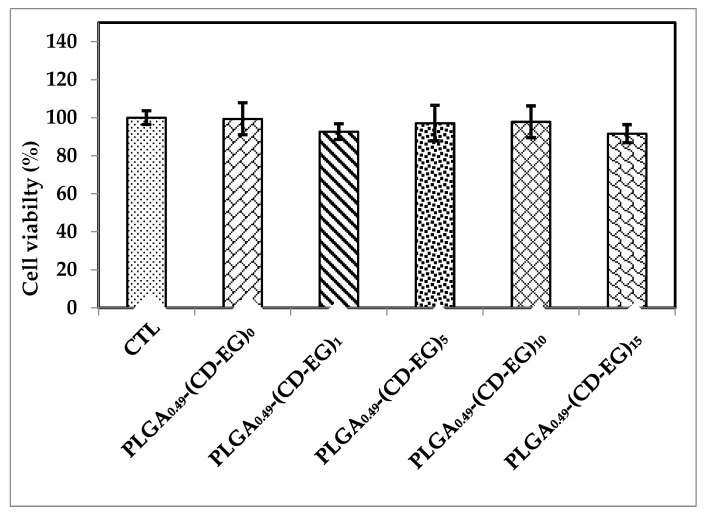
Cell viability of PLGA_0.49_-(CD-EG)_15_ at a concentration of 1 (µm/mL) on BV-2 microglial cells.

After preparation and characterization, the resulting samples were tested by MTT assays to their effects on cell viability of microglial cells BV-2 at 1 mg/mL). The results in Figure 6 showed cell viability of various formulations of PLGA microspheres. As observed, various formulations of PLGA_0.49_-CD-EG did not influence on microglial cells BV-2 at 1 mg/mL, as compared with controls. Therefore, it proved that PLGA_0.49_-CD-EG at a concentration of 1 mg/mL is safe to BV-2 microglial cells.

### 3.8. Investigation of NO Production

**Figure 7:**
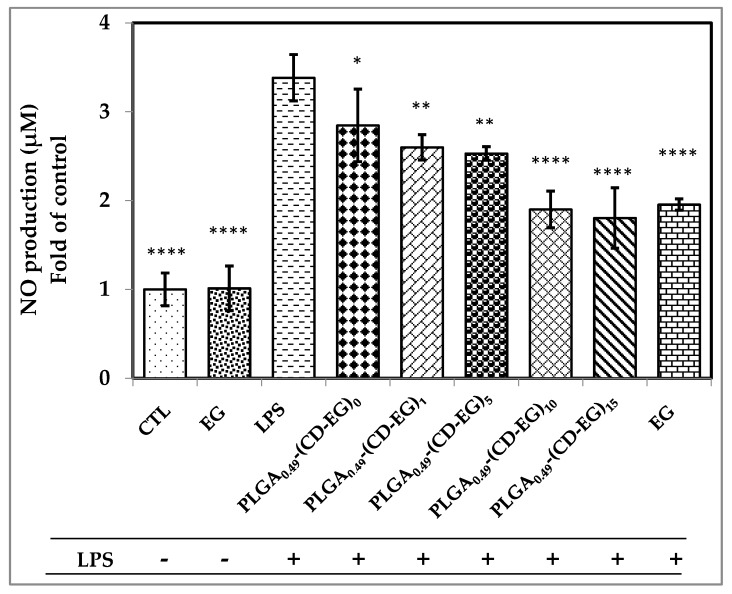
Effects of PLGA_0.49_-(CD-EG) formulations on NO production by BV-2 microglial cells in the presence or absence of 50 ng/mL of LPS by Griess reagent method. CTL is the control without the addition of EG and LPS. As compared to LPS, the marks of statistical significant *, **, *** and **** represent *p* < 0.05, 0.01, 0.001, and 0.0001, respectively.

Nitric oxide (NO) was investigated as a bioactive free radical which is related to different physiological pathways in the brain and spinal cord. It was proved that NO radicals played a critical role of stroke and various neurodegenerative diseases. NO production was induced when microglial cells were activated by some factors, such as LPS. In this study, we investigated the effects of various PLGA0.49-(CD-EG) formulations on NO production from activated BV-2 microglial cells with or without 50 ng/mL of LPS. BV-2 microglial cells were pre-treated with PLGA_0.49_-(CD-EG) for 48 h and then exposed to LPS for 24 h. The result in Figure 7 show that free EG showed no effect of NO production in the absence of LPS as compared to the control (CTL). In addition, the result demonstrated that LPS induced the remarkable increase of NO production, up to 3.4-fold, as compared to the control. When activated BV-2 microglial cells were pretreated, the NO production gradually decreases from 2.84- to 1.80-fold, as compared to the control. Especially, PLGA_0.49_-(CD-EG)_10_ and PLGA_0.49_-(CD-EG)_15_ showed the significant decrease of NO production. In addition, in the presence of LPS, NO production of PLGA_0.49_-(CD-EG)_10_ (1.89-fold) and PLGA_0.49_-(CD-EG)_15_ (1.80-fold) were lower than that of free EG (1.95-fold). Therefore, it proved that EG-CD complexes loaded in PLGA microspheres could suppress NO production in activated BV-2 cells as compared with free EG. As such, the result shows that PLGA_0.49_-(CD-EG)_15_ is the most effective formulation to suppress the inflammation of BV-2 cells in the present study.

### 3.9. Western Blot of iNOS in BV-2 Microglial Cells

Inducible nitric oxide synthase (iNOS) is a family of enzymes that catalyze the production of the free radical nitric oxide (NO), which is critical for cell signaling and cyto-protective events. At high and unregulated production of NO, it can provoke neurotoxicity in brain tissues [52]. Therefore, the regulation of NO production is important for maintaining NO-levels within physiological levels. In this study, Western blot was carried out to determine the level of iNOS proteins in BV-2 cells after treating with PLGA_0.49_-(CD-EG) microspheres.

**Figure 8:**
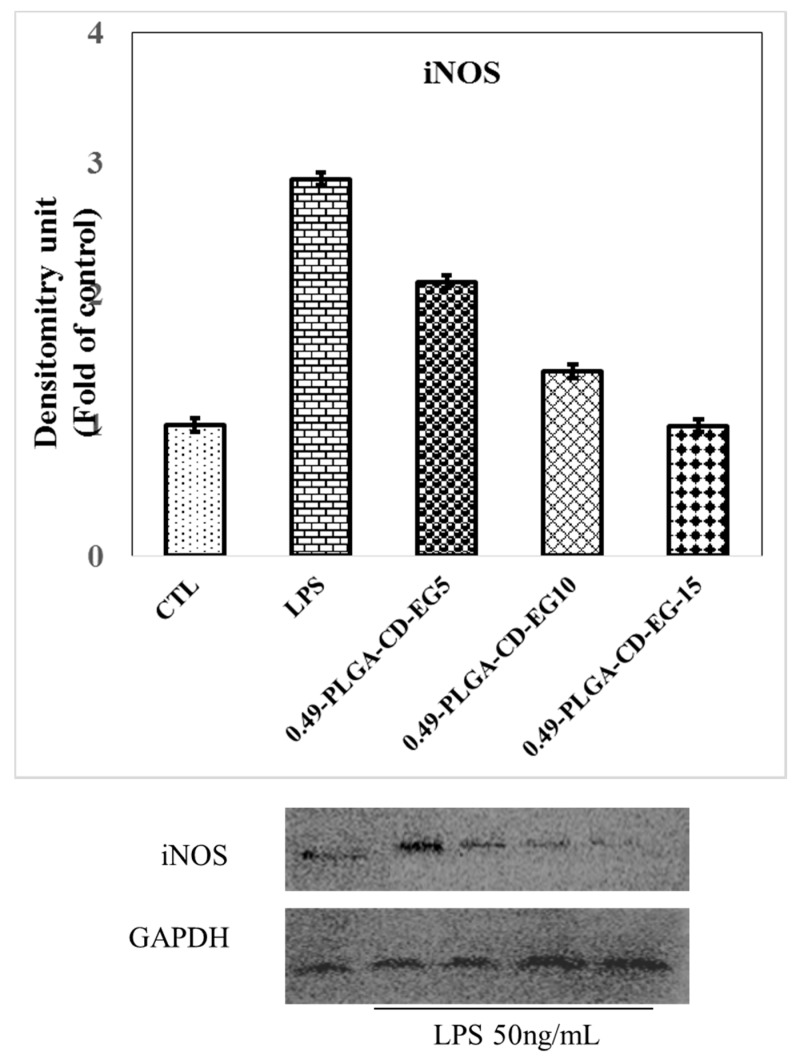
Effects of PLGA_0.49_-(CD-EG) formulations (1 mg/mL) on the expression of iNOS in BV-2 cells treated with 50 ng/mL of LPS for 24 h. Cultures were pretreated with PLGA_0.49_-(CD-EG) for 24 h before the the addition of LPS. Bars represent the mean ± standard errors from three independent experiments. Densitometry analyses are presented as the relative ratio of protein/GADPH protein and are represented as fold changes with respect to the control.

Figure 8 shows the iNOS levels in BV-2 cells activated by 50 ng/mL of LPS for 24 h. It was found that the iNOS levels significantly decreased after treating the cells with increased EG loadings of PLGA_0.49_-(CD-EG) microspheres. Specifically, the expression of iNOS remarkably dropped from 2.88-fold (LPS group) to 0.99-fold for PLGA_0.49_-(CD-EG)_15_ microspheres. In other words, PLGA_0.49_-(CD-EG)_15_ microspheres could maintain a basal (control) level of iNOS in microglia cells. This preliminary result proved the cytoprotective effect of PLGA_0.49_-(CD-EG)_15_ microspheres by regulating iNOS expression and reducing NO production as well. Therefore, the PLGA microspheres fabricated by the modified double-emulsion technique in this study is a potential micro-carrier to deliver EG to suppress the inflammation of BV-2 microglial cells activated by LPS.

The present study has demonstrated an increase in the drug loading of EG in PLGA microspheres through the complexation of β-CD. According to the release profile, the accumulative concentration for PLGA-CD-EG microspheres was significantly higher than that of PLGA-EG microspheres where no β-CD was used. The medical benefit of such a formulation is to exert neuroprotective effects in patients bearing Parkinson’s disease [25]. Other than the neuroprotective benefit, polyphenols such as EG was also reported to avoid gastrointestinal damage of the nonsteroidal anti-inflammatory drugs (NSAID) [53]. Therefore, the natural polyphenol, EG as the medication for Parkinson’s disease has two attributes, including anti-inflammation and non-damage to the gastric mucosa.

## 4. Conclusions

Various PLGA microspheres loading CD-EG complexes were successfully prepared using he double-emulsion evaporation method. The formation of CD-EG complexes was demonstrated using UV–Vis spectroscopy. The EG:β-CD ratios were optimized as of 1:1 to form CD-EG complexes. Apart from that, the results showed that, among various formulations, PLGA_0.49_-CD-EG showed the highest loading efficiency, with a value of 16.34%. In addition, it showed that the maximum amount of initial EG feeding was optimized at a feeding of EG of 15 mg.

Physicochemical properties of PLGA_0.49_-(CD-EG) microspheres were characterized by FTIR and SEM with spherical morphology and particles ranging in size from 1–14 µm. This result indicated that the maximum EG release from PLGA_0.49_-(CD-EG)_15_ were 261.17 µg/mL for a release time of 168 h. MTT assays showed that PLGA_0.49_-(CD-EG) did not influence on BV-2 microglial cell viability. In addition, the NO production result proved that PLGA_0.49_-(CD-EG)_15_ can inhibit the inflammation of microglial cells in the presence of LPS. Moreover, PLGA_0.49_-(CD-EG)_15_ was proved to inhibit the expression of iNOS in the intracellular environment of BV-2 cells. Finally, PLGA microspheres were proved to be promising carriers to improve the loading efficiency of EG in the presence of β-CD to suppress the inflammation of activated BV-2 cells.

## Figures and Tables

**Table 1:** Epigallocatechin-3-gallate reacts with β-cyclodextrin at various ratios to form CD-EG complexes.

Abbreviation	EG (µM)	β-CD (µM)
CD_80_EG_20_	80	20
CD_60_EG_40_	60	40
CD_50_EG_50_	50	50
CD_40_EG_60_	40	60
CD_20_EG_80_	20	80

**Table 2:** Abbreviations of the various PLGA microspheres with different initial amounts of CD-EG, BSA-EG, or EG. In this study, the molar ratio of CD to EG or BSA to EG in the CD-EG or BSA-EG complexes used was 1:1.

Feeding CD-EG, BSA-EG or EG (mg)	PLGA Copolymers			
	PLGA_0.85_	PLGA_0.85_	PLGA_0.53_	PLGA_0.49_
0	N/A	N/A	N/A	PLGA_0.49_-(CD-EG)_0_
1	PLGA_0.85_-EG_1_	PLGA_0.85_-(BSA-EG)_1_	PLGA_0.53_-(CD-EG)_1_	PLGA_0.49_-(CD-EG)_1_
5	PLGA_0.85_-EG_5_	PLGA_0.85_-(BSA-EG)_5_	PLGA_0.53_-(CD-EG)_5_	PLGA_0.49_-(CD-EG)_5_
10	PLGA_0.85_-EG_10_	PLGA_0.85_-(BSA-EG)_10_	PLGA_0.53_-(CD-EG)_10_	PLGA_0.49_-(CD-EG)_10_
15	PLGA_0.85_-EG_15_	PLGA_0.85_-(BSA-EG)_15_	PLGA_0.53_-(CD-EG)_15_	PLGA_0.49_-(CD-EG)_15_

**Table 3:** Drug loading efficiency (%) of various PLGA copolymers with different feedings of CD-EG, BSA-EG, or EG.

Formulation	Feeding of CD-EG, BSA-EG or EG			
	1 mg	5 mg	10 mg	15 mg
PLGA_0.85_EG	0.26	0.32	1.35	2.06
PLGA_0.85_-(BSA-EG)	1.07	1.82	2.65	3.24
PLGA_0.53_-(CD-EG)	2.64	3.65	8.02	9.69
PLGA_0.49_-(CD-EG)	2.68	3.81	9.12	16.34
